# A Bayesian approach for parameter estimation in the extended clock gene circuit of Arabidopsis thaliana

**DOI:** 10.1186/1471-2105-14-S10-S3

**Published:** 2013-08-12

**Authors:** Catherine F Higham, Dirk Husmeier

**Affiliations:** 1 School of Mathematics and Statistics, College of Science and Engineering, University of Glasgow, Glasgow G12 8QQ, Scotland, UK

## Abstract

The circadian clock is an important molecular mechanism that enables many organisms to anticipate and adapt to environmental change. Pokhilko *et al*. recently built a deterministic ODE mathematical model of the plant circadian clock in order to understand the behaviour, mechanisms and properties of the system. The model comprises 30 molecular species (genes, mRNAs and proteins) and over 100 parameters. The parameters have been fitted heuristically to available gene expression time series data and the calibrated model has been shown to reproduce the behaviour of the clock components. Ongoing work is extending the clock model to cover downstream effects, in particular metabolism, necessitating further parameter estimation and model selection. This work investigates the challenges facing a full Bayesian treatment of parameter estimation. Using an efficient adaptive MCMC proposed by Haario *et al*. and working in a high performance computing setting, we quantify the posterior distribution around the proposed parameter values and explore the basin of attraction. We investigate if Bayesian inference is feasible in this high dimensional setting and thoroughly assess convergence and mixing with different statistical diagnostics, to prevent apparent convergence in some domains masking poor mixing in others.

## Introduction

**Figure 1:**
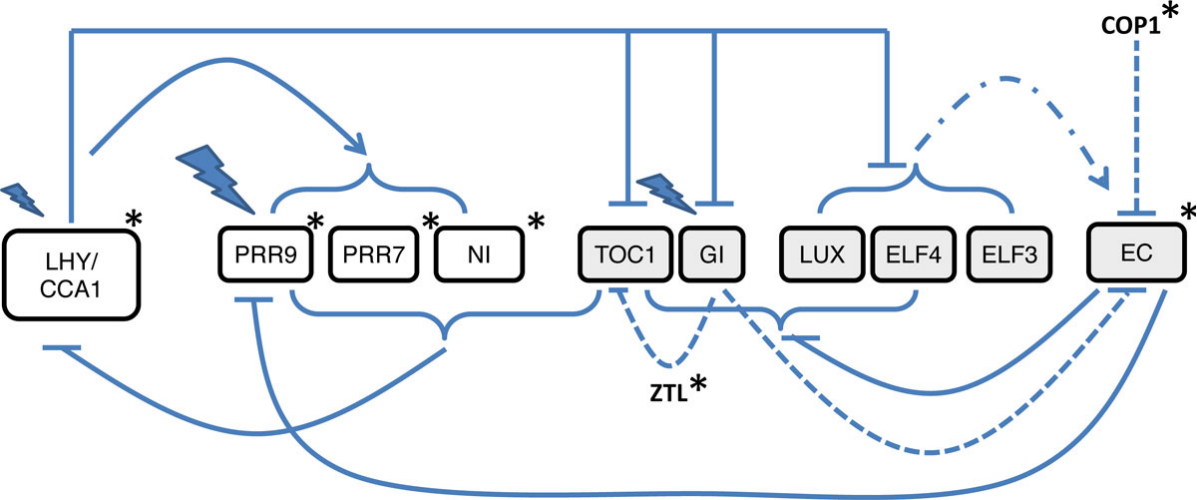
**Outline of the Arabidopsis circadian clock based on Figure 1 in **[4]. The morning and evening loop elements are represented by white and grey boxes respectively. The solid lines indicate transcriptional regulation and the short dashed lines indicate post-translational regulation of TOC1 and EC by GI, ZTL and COP1. An arrow signifies activation and a block inhibition. The EC protein complex formation is denoted by the short-long dashed line. Flashes represent acute light responses and asterisks post-translational regulation by light.

The circadian clock is a molecular mechansism that synchronises biological processes with the day/night cycle and is found in many organisms [1]. The presence of a clock enables an organism to anticipate and adapt to environmental change and hence use energy sources more efficiently. The mechanism includes interlocked, transcriptional feedback loops. Pokhilko *et al*. built a mathematical model of the plant circadian clock in order to understand the behaviour, mechanisms and properties of the system [2]. Recent experiments show that three plant-specific proteins ELF3, ELF4 and LUX form an evening complex (EC) which binds to the promoters of target genes [3]. This has led to a revision of the model [4]. The latest version of the clock model, illustrated in Figure 1, represents interconnected morning and evening loops in a three loop structure. The morning loop comprises transcription factors *LHY *and *CCA1*, which activate the expression of *PRR9 PRR7 *and *PRR5/NI*. The transcriptional co-regulators PRR9, PRR7 and PRR5 inhibit *LHY *and *CCA1 *expression by binding to their promoters. The evening loop, previously represented by TOC and a hypothetical gene (Y), introduced by Locke *et al. *[5] is now represented by TOC, LUX, ELF3 and ELF4.

Parameter values used in these studies were either constrained (based on the available data) or fitted to multiple time series data sets (for full details see Supplementary Table S1 in [4]). Previously, Locke *et al*. constructed a cost function to quantify the agreement between an earlier version of the model and various key experimental features [5]. They undertook an efficient global search of parameter space and showed that this optimized solution fits several but not all the experimental features.

The aim of this work is to set the scene for full Bayesian inference of the model parameters using state-of-the-art MCMC techniques. We explore the landscape of the posterior distribution around the model solution and investigate the challenges in finding the posterior distribution from increasingly distant initial starting positions in parameter space. We consider the implications of our findings on the design of efficient parameter estimation schemes for components of the circadian clock.

## Methods

### Mathematical model and data

We apply the methods to synthetic data generated from the model ODEs, described by Pokhilko *et al. *[4] where the actual parameter values are known. We use the latest version of the model which comprises the evening complex and a light function mimicking 12 hours day and 12 hours night. The model comprises 28 species (i.e. mRNAs and proteins) and an additional two species are used for fitting purposes, in total 30 species. There are 103 unknown parameters. Typical differential equations for the dimensionless concentrations of ELF4 mRNA, $c_{E4}^{m}$, suppressed by EC and LUX proteins, *c_EC _*and *c_LUX _*, respectively; and EFL4 protein, *c*_*E*4_, modified by ELF3 nuclear protein, *c*_*E*3*n*_, and ELF3-ELF4 nuclear protein complex, *c*_*E*34*n*_, are:

(1) $$\frac{dc_{E4}^{m}}{dt}=n_{13}\cdot \frac{g_{2}}{g_{2}+c_{EC}}\cdot \frac{g_{6}^{2}}{g_{6}^{2}+c_{L}^{2}}-m_{34}c_{E4}^{m},$$

(2) $$\frac{dc_{E4}}{dt}=p_{23}c_{E4}^{m}-m_{35}c_{E4}-p_{25}c_{E4}c_{E3n}+p_{21}c_{E34n}$$

where *n*_13_, *m*_34,35_, *p*_21,23,25 _and *g*_2,6 _represent the rate constants of transcription, degradation, protein translation /modification /complex formation /translocation between nucleus and cytoplasm and Michaelis-Menton constants, respectively. The full set of differential equations can be found in the supplementary material in Pokhilko et al. [4].

The use of synthetic data in this way enables us to assess the accuracy of the inference prediction. White Gaussian noise was added to this time series data to obtain a signal to noise ratio (SNR) of around 100. Fifty time points, one every hour over a two day period was considered realistic and suitably rich to capture key features of the data, namely the oscillatory period, amplitude and phase.

### Parameter estimation

We derived posterior distributions for the model parameters under a Bayesian framework using the efficient adaptive Markov chain Monte Carlo (MCMC) algorithm described by Haario *et al*., implemented using their MATLAB code [6]. This method uses a multivariate Gaussian proposal to move the exploratory chains through posterior spaces which may contain ridges or other challenging features - very likely when, as in our case, the number of parameters is high and/or parameters are correlated.

The algorithm described above requires a model defined sum-of-squares function and assumes additive i.i.d. Gaussian errors for the observations. Letting *ψ *(*θ*) be the ODE solution for a set of initial conditions and parameters, *θ*, then, in our case, the sum of squares function is

(3) $$\overset{N}{\underset{n=1}{\sum }}{\left(\psi_{n}\left(\theta \right)-y_{n}\right)}^{2}$$

where *ψ*_n_(*θ*) is the model output corresponding to the *n*th data point, _yn_. This score is equivalent to the negative log likelihood under a homoscedastic additive i.i.d. Gaussian noise model [7]. For the prior, we assumed an improper uniform distribution. This is the worst-case scenario corresponding to the complete absence of complementary biological information, which was chosen deliberately so as to obtain a conservative lower bound on both the parameter estimation accuracy as well as the rate of convergence.

The MCMC chains should be run until they have satisfactorily converged. A suitable number of steps is not typically known in advance and hence convergence diagnostics are used to monitor convergence. Our chains were run for approximately 10^6 ^iterations. Four sub chains were used to establish the potential scale reduction factor (PSRF). The PSRF is a ratio reflecting the between chain variance and the within chain variance. This and other methods are discussed in [8]. If the PSRF is large then either our estimate of variance can be decreased by more simulations or the within chain variance will increase since the simulated sequences have not yet made a full tour of the target distribution. Generally a value of 1.1 is taken to indicate reasonable confidence that the chains have converged. PSRF is estimated in parameter space but we also use it to consider whether the sum-of-squares function, equation 3 has converged in data space. The multivariate analogue (MPSRF), essentially an upper bound of PSRF, was used to consider convergence at the higher order [8]. Posterior measures, including mean and variance are based on every 100th iteration of the last 10^5 ^iterations, pruned to reduce levels of autocorrelation.

Obtaining the numerical solution of the ODE, *ψ_n_*(*θ*), is an expensive component of the overall computational task so we accelerated its calculation using high speed ODE simulations available from SBTOOLBOX2 and SBPD [9]. On average this made the calculations between 10 and 20 times faster than those based on MATLAB's built-in ODE solvers. Computations were designed to run on a multi-nodal cluster using MATLAB's parallelization facilities.

## Results

### Quantification of parameter posteriors starting at true parameter values

In our first test, denoted Experiment 1, we start the MCMC exploratory chains at the model's true values and observe how far the chains will travel before they reach a stationary phase. The Euclidean distance between the posterior mean and the true value, for each parameter, is indicative of the attractive pull of the model solution. The variance of the posterior distribution summarizes the inherent uncertainty in the system. Noise in the system, arising from measurement errors, may mean that the maximum *a posteriori *does not lie directly over the true value.

After 10^6 ^iterations, the sum-of-squares plots, tracing the fit of the model to the data, are indicated to have converged (mean PSRF = 1.01), see Table 1. However the multivariate statistic (MPSRF = 1.81) indicates that the trace plots are still changing with implications for the accuracy of the fit of the model between species. Concerning parameter space, 99% of parameters show no absence of convergence as indicated by PSRF*<*1.1 (mean PSRF = 1.02) and all the posterior estimates lie within the 5*^th^*-95*^th ^*percentile of the posterior distribution. The Euclidean distance in parameter space between the MCMC chain start and the true value (denoted *ED*_0_) is zero and the Euclidean distance between the posterior mean and the true value is 0.25 (denoted *ED*_m_). This is our first indication of how far it is possible to perturb the parameters before they lie outside the basin of attraction.

**Table 1:** Summary of convergence diagnostics in parameter and data space

Experiment	Euclidean distance from true values at start ED_0_	Species Analysis PSRF computed from sum-of-squares trace plots in data space			Parameter Analysis PSRF computed from MCMC chains in parameter space			Euclidean distance between true values and posterior mean ED_m_	%True parameter values lying outside 5^th ^- 95^th ^percentile of posterior distribution
				
		%Species PSRF <1.1	Mean PSRF	MPSRF	%Parameters PSRF <1.1	Mean PSRF	MPSRF		
1. No perturbation	0	100	1.01	1.81	99	1.02	2.69	0.25	0

2. Perturbed variance = 0.005^2^	0.06	100	1.01	1.77	95	1.03	3.38	0.30	0.8

3. Perturbed variance = 0.01^2^	0.12	97	1.01	1.80	90	1.03	3.07	0.22	1.5

4. Perturbed variance= 0.015^2^	0.20	97	1.01	1.85	92	1.03	3.68	0.30	1.5

5. Perturbed variance= 0.02^2^	0.24	97	1.02	2.12	96	1.03	3.47	0.33	0.8

6. Perturbed variance= 0.025^2^	0.30	93	1.03	2.11	68	1.09	3.37	0.32	3.0

7. Perturbed variance = 0.03^2^	0.32	100	1.01	1.74	100	1.01	2.85	0.43	3.8

8. Perturbed variance = 0.035^2^	0.38	87	1.05	2.50	62	1.11	3.82	0.37	6.8

9. Perturbed variance = 0.05^2^	0.51	57	1.10	2.26	45	1.21	3.48	0.49	10.5

10. Perturbed variance = 0.1^2^	0.98	33	1.35	4.47	24	1.41	5.79	0.59	11.3

11. Gamma(1,2)	87.66	7	1.98	8.47	18	2.14	10.29	0.70	21.1

12. Gamma(2,4)	39.86	7	2.31	9.14	5	2.04	10.73	16.03	21.1

13. Gamma(2,4)	88.12	3	2.36	5.81	2	1.82	6.89	40.90	15.8

### Perturbance of the starting position further defines attraction of the posterior basin

Next, we perturbed the starting point of the exploratory adaptive MCMC chains by sampling from a Normal distribution centred on the true value with variance increasing in small steps from variance= 0.005^2 ^(corresponding to *ED*_0 _= 0.06) to variance= 0.1^2 ^(*ED*_0 _= 0.98). We denote these experiments as Experiments 2 to 10, see Table 1. We then tried choosing initial starting parameters from a Gamma distribution, Γ (1, 2) and Γ (2, 4), denoted Experiments 11 to 13. For all but two experiments where variance were less than 0.015^2 ^(Experiments 1 to 3) we took the initial start from inferring the initial condition to be the observed level in our data, see Table 1.

Generally as the initial starting value is perturbed the mean PSRF value for the sum-of-squares trace plots increases and the number of species converging reduces (Columns 3 and 4, Table 1). Except for the first three experiments, with no or little perturbation, *ED_m _*(Column 10, Table 1) is comparable with the *ED*_0 _(Column 2, Table 1), suggesting that the MCMC parameter chains are not venturing far from the true values. However by Experiment 9 the perturbation is such that over 10% of the parameters are converging to posterior means that are significantly far away from the true value (i.e. true value lies outside the 5*^th^*-95*^th ^*percentile posterior distribution). This could be indicative of alternative parameter regimes giving rise to the observed data and can be tested by inspecting the log likelihood.

### Parameter convergence is not correlated to Euclidean distance between starting value and true value

**Figure 2:**
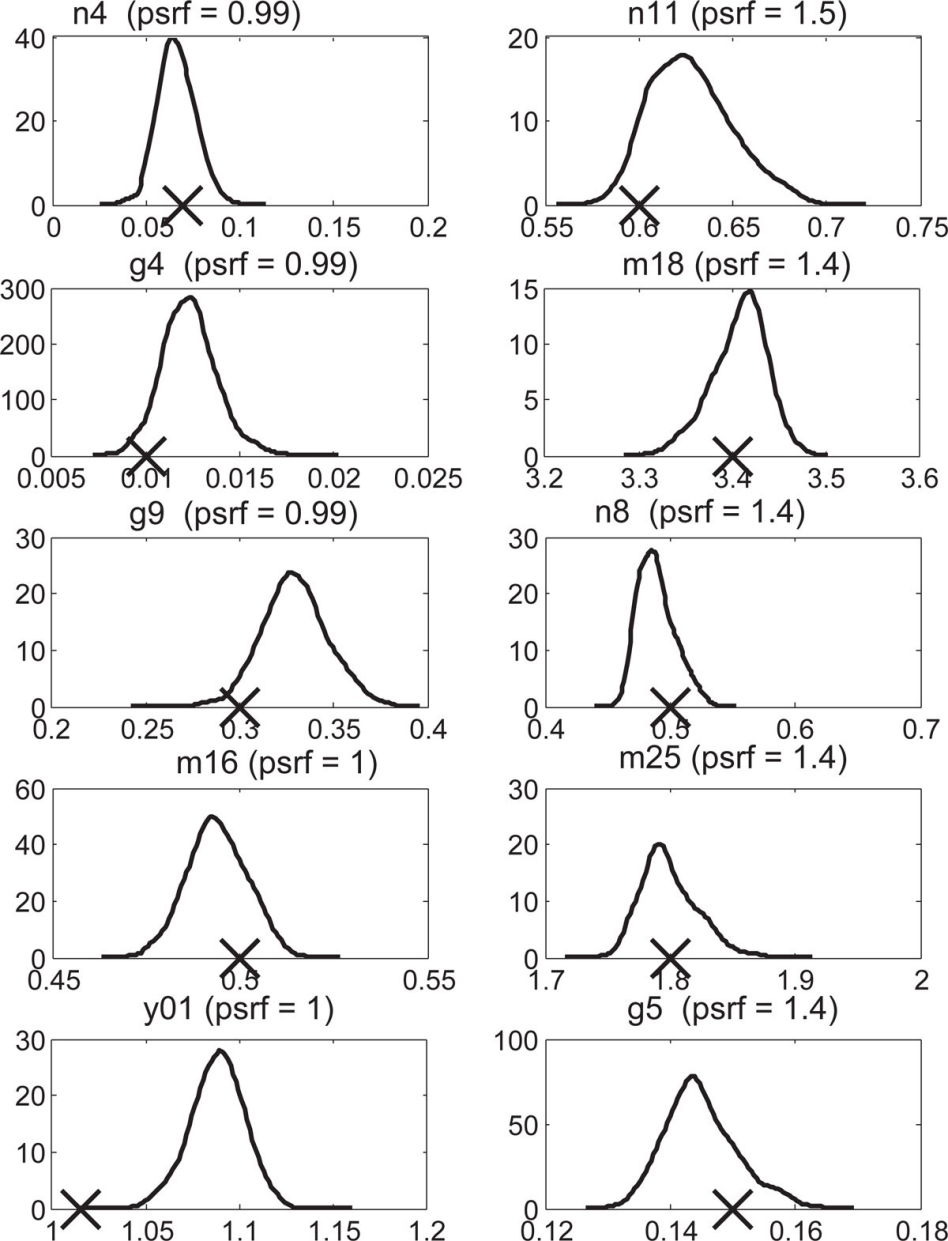
**Posterior probability density estimates for parameters with low end PSRFs (left column) and parameters with high end PSRFs (right column)**. True parameter values are indicated by a cross on the x-axis.

As mentioned above, convergence in data space at the species level decreases with distance, most notably as *ED*_0 _crosses *ED*_m_. Hence we checked whether convergence in parameter space was correlated to initial start values. We found that neither *ED*_0 _nor the percentage of *ED*_0 _to the true value is significantly correlated (*P >*0.05) with the PSRF for Experiments 1-7. Examination of the marginal posterior distributions for the five parameters with the lowest PSRFs and the five highest in Experiment 6 (Figure 2) illustrates that recovery of the true parameter values may not be controlled directly by convergence diagnostics.

The reason why some parameters are not converging appears parameter specific and possibly related to individual levels of inter-correlation with other parameters or species. To test this hypothesis we investigated what happens if we remove highly correlated parameters from the analysis. We saw a reduction in the MPSRF (e.g. removing parameters with a correlation coefficient of 0.25 or above would reduce the MPSRF from 3.38 to 1.02 in Experiment 2) but more work will be required to systematically expose patterns of inter-correlations in this complex network of 30 species and over 100 parameters.

## Conclusions

The aim of this work is to set the scene for full parameter estimation and model comparison in a Bayesian context for the circadian clock model. Previously, model parameters have been fitted heuristically [2,4,10]. Those model calibration exercises reproduce features of the data but cannot rule out other parameter regimes. As the clock continues to be extended to other species and to downstream activities such as metabolism it becomes increasingly important to evaluate competing scenarios, and the Bayesian approach extends naturally to model comparison. This work represents the first full Bayesian treatment of parameter estimation for the circadian clock in *Arabidopsis Thaliana *and informs future work for tackling this complex problem.

Our initial investigation highlights two main areas. First, we have shown that modern MCMC techniques, when implemented in a high performance computing environment, make it feasible to attempt Bayesian inference in this high-dimensional setting.

Second, apparent convergence in either data or parameter space, using diagnostic techniques, may mask poor mixing, both pairwise and at higher orders of the exploratory chains. This issue requires further investigation of the proposal function but also better coverage of the prior parameter space with a population of chains. For the simulation experiments described in the present article, we took the most unfavourable scenario of complete absence of prior information about the chemical kinetic parameter values, for which we chose improper uniform prior distributions. For most practical applications, more informative priors are usually available, derived from expert elicitation, the biological literature, databases such as KEGG (Kyoto Encyclopedia of Genes and Genomes), and complementary experiments. We note that more informative priors can not only potentially lead to an improvement in the parameter estimation accuracy, but also to an improvement in the convergence of the Markov chains, due to the fact that they render the posterior distributions less diffuse. The estimates presented in the present study can therefore be regarded as conservative, providing performance indicators that, in practice, can potentially be improved on. Exploration of the parameter space could also be directed by introducing auxiliary information, in a systematic fashion, specific to the circadian model, such as period/amplitude or phase of the data. These considerations will allow for greater confidence in the predictions and fuller understanding of the model performance in different parameter regimes.

## Competing interests

The authors declare that they have no competing interests.

## Authors' contributions

CFH designed and executed the computer experiments. CFH and DH wrote the manuscript.

## Declarations

The publication costs for the article are covered by the TiMeT project.

This article has been published as part of BMC Bioinformatics Volume 14 Supplement 10, 2013: Selected articles from the 10th International Workshop on Computational Systems Biology (WCSB) 2013: Bioinformatics. The full contents of the supplement are available online at http://www.biomedcentral.com/bmcbioinformatics/supplements/14/S10
